# Supplementary material for: Identification of Somatic Mutations in Plasma Cell-Free DNA from Patients with Metastatic Oral Squamous Cell Carcinoma
Source: Int J Mol Sci. 2023 Jun 20;24(12):10408. doi: 10.3390/ijms241210408 (PMC10298855; doi:10.3390/ijms241210408)
Supplement: Supplementary file 1 [file ijms-24-10408-s001.zip › ijms-2414300-supplementary.pdf]

Supplementary Materials

Table S1. Unadjusted and adjusted linear regression predicting change in tumor mutation burden

	Unadjusted analysis			Adjusted analysis[a]		
Parameter	B	95% CI	*P*-value	B	95% CI	*P*-value
Clinical stage (I-III)						
IV	0.157	-9.03-30.99	0.275	0.153	-9.63-30.94	0.296
Metastasis (No)						
Yes	0.312	2.71-44.22	0.028	0.320	3.13-45.07	0.025

95% CI, 95% confidence interval

B, standardized coefficients

[a]Regression models adjusted by age

Table S2. List of most frequently mutated genes in TCGA-OSCC dataset

Genes	Patients	(%)
TP53	264	(68%)
TTN	163	(42%)
FAT1	99	(26%)
CDKN2A	86	(22%)
MUC16	77	(20%)
NOTCH1	75	(19%)
PIK3CA	69	(18%)
LRP1B	61	(16%)
SYNE1	59	(15%)
PCLO	58	(15%)
DNAH5	55	(14%)
FLG	53	(14%)
CSMD3	53	(14%)
DST	51	(13%)
CASP8	51	(13%)
PLEC	51	(13%)
KMT2D	49	(13%)
AHNAK	46	(12%)
HUWE1	44	(11%)
USH2A	42	(11%)
XIRP2	40	(10%)
FAT4	38	(10%)
MUC5B	38	(10%)
SYNE2	36	(9%)
MACF1	36	(9%)
OBSCN	35	(9%)
RYR2	35	(9%)
AHNAK2	35	(9%)
MUC17	35	(9%)
DMD	34	(9%)
RELN	34	(9%)
PCDH15	33	(9%)
EP300	33	(9%)

Table S3. List of most frequently mutated genes in our cfDNA and TCGA-OSCC dataset

Genes	Our study	TCGA-OSCC	Genes	Our study	TCGA-OSCC
	Patients (%)	Patients (%)		Patients (%)	Patients (%)
TTN	24 (48%)	163 (42%)	CFAP46	16 (32%)	7 (2%)
PLEC	23 (46%)	51 (13%)	FAT4	16 (32%)	38 (10%)
SYNE1	22 (44%)	59 (15%)	CACNA1B	16 (32%)	14 (4%)
RYR3	22 (44%)	28 (7%)	DOCK10	16 (32%)	6 (2%)
DMD	20 (40%)	34 (9%)	POLE	16 (32%)	8 (2%)
HECTD4	20 (40%)	19 (5%)	SBNO2	16 (32%)	6 (2%)
UBR4	19 (38%)	19 (5%)	ZAN	16 (32%)	15 (4%)
KMT2D	19 (38%)	49 (13%)	DOCK2	16 (32%)	12 (3%)
RYR1	19 (38%)	27 (7%)	OTOF	16 (32%)	11 (3%)
MYCBP2	19 (38%)	20 (5%)	ABCC1	16 (32%)	11 (3%)
NBEAL1	19 (38%)	11 (3%)	HERC1	15 (30%)	18 (5%)
NEB	18 (36%)	31 (8%)	TNXB	15 (30%)	16 (4%)
OBSCN	18 (36%)	35 (9%)	SPEN	15 (30%)	22 (6%)
DNAH10	18 (36%)	18 (5%)	VPS13C	15 (30%)	19 (5%)
SYNE2	18 (36%)	36 (9%)	BIRC6	15 (30%)	18 (5%)
VPS13D	18 (36%)	29 (7%)	DNAH1	15 (30%)	14 (4%)
PKHD1L1	18 (36%)	30 (8%)	DYNC1H1	15 (30%)	23 (6%)
ZFHX4	18 (36%)	30 (8%)	MDN1	15 (30%)	21 (5%)
LRP1B	18 (36%)	61 (16%)	ABCA13	15 (30%)	27 (7%)
USH2A	18 (36%)	42 (11%)	MUC5AC	15 (30%)	17 (4%)
SACS	18 (36%)	23 (6%)	DNAH2	15 (30%)	24 (6%)
KIAA1109	17 (34%)	22 (6%)	ABCA2	15 (30%)	11 (3%)
DNAH9	17 (34%)	31 (8%)	AKAP9	15 (30%)	21 (5%)
TRRAP	17 (34%)	18 (5%)	ANK3	15 (30%)	16 (4%)
DNAH5	17 (34%)	55 (14%)	ARFGEF3	15 (30%)	8 (2%)
CCDC168	17 (34%)	#N/A	COL11A1	15 (30%)	30 (8%)
EP400	17 (34%)	14 (4%)	KALRN	15 (30%)	21 (5%)
FLNA	17 (34%)	19 (5%)	COL12A1	15 (30%)	8 (2%)
DNAH11	17 (34%)	28 (7%)	DNHD1	15 (30%)	8 (2%)
FRAS1	17 (34%)	21 (5%)	HSPG2	15 (30%)	19 (5%)
LRP1	17 (34%)	26 (7%)	CSMD2	15 (30%)	19 (5%)
SRRM2	17 (34%)	14 (4%)	DCHS1	15 (30%)	13 (3%)
PRKDC	17 (34%)	21 (5%)	DNAH7	15 (30%)	17 (4%)
ZZEF1	17 (34%)	12 (3%)	KMT2C	15 (30%)	23 (6%)
MGA	17 (34%)	11 (3%)	WDFY3	15 (30%)	14 (4%)
RNF213	17 (34%)	27 (7%)	CACNA1D	15 (30%)	15 (4%)
ATM	17 (34%)	15 (4%)	COL4A6	15 (30%)	10 (3%)

PLXNA3	17 (34%)	17 (4%)	IGFN1	15 (30%)	5 (1%)
ARID1A	17 (34%)	14 (4%)	PLXND1	15 (30%)	7 (2%)
FBN3	17 (34%)	11 (3%)	COL5A3	15 (30%)	10 (3%)
KNTC1	17 (34%)	13 (3%)	SEC16A	15 (30%)	10 (3%)
DOCK7	17 (34%)	11 (3%)	SHANK1	15 (30%)	8 (2%)
TRPM6	17 (34%)	13 (3%)	TRANK1	15 (30%)	12 (3%)
ANK1	17 (34%)	9 (2%)	CNOT1	15 (30%)	15 (4%)
NFAT5	17 (34%)	8 (2%)	INTS1	15 (30%)	3 (1%)
ANK2	16 (32%)	18 (5%)	PREX1	15 (30%)	11 (3%)
SSPO	16 (32%)	18 (5%)	BCOR	15 (30%)	4 (1%)
ADGRV1	16 (32%)	24 (6%)	DEPDC5	15 (30%)	7 (2%)
MACF1	16 (32%)	36 (9%)	KIDINS220	15 (30%)	6 (2%)
FAT3	16 (32%)	31 (8%)	MAP1B	15 (30%)	16 (4%)
ADGRG4	16 (32%)	21 (5%)	COL4A4	15 (30%)	7 (2%)
RYR2	16 (32%)	35 (9%)	CREBBP	15 (30%)	27 (7%)
HMCN1	16 (32%)	26 (7%)	KIAA1551	15 (30%)	8 (2%)
STARD9	16 (32%)	#N/A	COL22A1	15 (30%)	27 (7%)
TG	16 (32%)	18 (5%)	CRAMP1	15 (30%)	#N/A
LAMA2	16 (32%)	18 (5%)	SHROOM2	15 (30%)	3 (1%)
NSD1	16 (32%)	27 (7%)	CELSR1	15 (30%)	16 (4%)
UNC80	16 (32%)	5 (1%)	GBF1	15 (30%)	5 (1%)
FBN2	16 (32%)	16 (4%)	MUC5B	15 (30%)	38 (10%)
KMT2A	16 (32%)	23 (6%)	NCOR2	15 (30%)	13 (3%)
LYST	16 (32%)	13 (3%)	NOTCH1	15 (30%)	75 (19%)
HMCN2	16 (32%)	#N/A	PKD1L2	15 (30%)	13 (3%)

Table S4. The relationship between metastatic status and mutated genes

Parameters		Non-metastasis (N=31)	Metastasis (N=9)	*P*-value[a]
TTN	Wide type	29	2	0.018
	Mutant	12	7	
SYNE1	Wide type	29	2	0.018
	Mutant	12	7	
RYR3	Wide type	30	2	0.007
	Mutant	11	7	
DMD	Wide type	29	2	0.018
	Mutant	12	7	
HECTD4	Wide type	30	2	0.007
	Mutant	11	7	
KMT2D	Wide type	31	1	**0.001**
	Mutant	10	8	
NEB	Wide type	31	3	0.022
	Mutant	10	6	
DNAH10	Wide type	33	1	**<0.001**
	Mutant	8	8	
SYNE2	Wide type	33	3	0.009
	Mutant	8	5	
VPS13D	Wide type	32	2	0.003
	Mutant	9	7	
ZFHX4	Wide type	33	3	0.009
	Mutant	8	6	
LRP1B	Wide type	31	3	0.022
	Mutant	10	6	

[a]*P*-value were determined with Fisher's exact test

The Bonferroni-adjusted threshold for significance is set at α = 0.002 (0.05/33)

Table S5. List of the 300 most frequent gene

Gene	Mutations	Patietns (%)	Gene	Mutations	Patietns (%)	Gene	Mutations	Patietns (%)
MUC16	111	23 (48%)	DNAH7	30	15 (30%)	ANKRD24	15	13 (26%)
TTN	116	24 (48%)	GBF1	22	15 (30%)	APC	15	13 (26%)
PLEC	51	23 (46%)	HERC1	54	15 (30%)	ARID1B	21	13 (26%)
SYNE1	87	23 (46%)	IGFN1	28	15 (30%)	ARMCX4	24	13 (26%)
RYR3	62	22 (44%)	IRS2	19	15 (30%)	ASCC3	21	13 (26%)
HECTD4	47	21 (42%)	KALRN	32	15 (30%)	ATP13A1	17	13 (26%)
KMT2D	62	21 (42%)	KIAA1551	24	15 (30%)	ATP7A	20	13 (26%)
OBSCN	78	21 (42%)	KIDINS220	25	15 (30%)	BOD1L1	21	13 (26%)
RYR1	51	21 (42%)	KMT2C	30	15 (30%)	BRWD1	19	13 (26%)
UBR4	57	21 (42%)	MAP1B	25	15 (30%)	BSN	29	13 (26%)
DMD	48	20 (40%)	MDN1	39	15 (30%)	BTAF1	17	13 (26%)
NBEAL1	26	20 (40%)	MUC5AC	35	15 (30%)	CACNA1F	25	13 (26%)
USH2A	37	20 (40%)	MUC5B	22	15 (30%)	CACNA1G	22	13 (26%)
LRP1	43	19 (38%)	NCOR2	22	15 (30%)	CFAP74	16	13 (26%)
LRP1B	44	19 (38%)	NOTCH1	22	15 (30%)	CGNL1	18	13 (26%)
MACF1	56	19 (38%)	PKD1L2	22	15 (30%)	CHD5	19	13 (26%)
MYCBP2	40	19 (38%)	PLXND1	28	15 (30%)	CHD7	24	13 (26%)
SYNE2	53	19 (38%)	PREX1	26	15 (30%)	CHD9	25	13 (26%)
ABCC1	24	18 (36%)	SEC16A	27	15 (30%)	COL6A3	27	13 (26%)
ATM	36	18 (36%)	SHANK1	27	15 (30%)	COL6A5	25	13 (26%)
DNAH1	50	18 (36%)	SHROOM2	23	15 (30%)	CSPG4	18	13 (26%)
DNAH10	44	18 (36%)	SPEN	40	15 (30%)	CUX1	20	13 (26%)
DNAH11	39	18 (36%)	TNXB	44	15 (30%)	DNAH14	20	13 (26%)
DNHD1	39	18 (36%)	TRANK1	27	15 (30%)	DNAH3	20	13 (26%)
FAT4	35	18 (36%)	USP25	21	15 (30%)	DOPEY2	32	13 (26%)
INTS1	30	18 (36%)	VPS13C	40	15 (30%)	DYSF	27	13 (26%)
MUC19	57	18 (36%)	WDFY3	30	15 (30%)	EML5	17	13 (26%)
MYO9B	26	18 (36%)	ABCA1	24	14 (28%)	ERBB4	16	13 (26%)
NEB	55	18 (36%)	ALMS1	28	14 (28%)	FAT2	26	13 (26%)
PKHD1L1	42	18 (36%)	ARFGEF2	27	14 (28%)	FBF1	16	13 (26%)
SACS	26	18 (36%)	BICRA	17	14 (28%)	FBN1	28	13 (26%)
VPS13D	44	18 (36%)	BPTF	22	14 (28%)	FMNL3	17	13 (26%)
ZFHX4	38	18 (36%)	C3	24	14 (28%)	FNIP1	18	13 (26%)
ANK1	22	17 (34%)	CDH23	37	14 (28%)	FRY	33	13 (26%)
ARID1A	32	17 (34%)	CEP350	30	14 (28%)	FRYL	25	13 (26%)
CCDC168	41	17 (34%)	CMYA5	30	14 (28%)	FYCO1	17	13 (26%)
CELSR1	34	17 (34%)	CSF3R	16	14 (28%)	GAPVD1	18	13 (26%)
DNAH5	42	17 (34%)	CSMD1	40	14 (28%)	GEMIN5	21	13 (26%)
DNAH9	44	17 (34%)	CTTNBP2	17	14 (28%)	GOLGA4	18	13 (26%)
DOCK7	24	17 (34%)	CUBN	27	14 (28%)	GOLGB1	25	13 (26%)
EP400	37	17 (34%)	CUL9	25	14 (28%)	HIVEP1	24	13 (26%)

FBN3	29	17 (34%)	DIP2A	24	14 (28%)	HKR1	16	13 (26%)
FLNA	37	17 (34%)	DNAH12	31	14 (28%)	HTT	25	13 (26%)
FRAS1	36	17 (34%)	DNAH17	30	14 (28%)	HUWE1	50	13 (26%)
KIAA1109	45	17 (34%)	DOT1L	21	14 (28%)	IGF2R	20	13 (26%)
KNTC1	28	17 (34%)	DSCAML1	21	14 (28%)	IQGAP3	16	13 (26%)
MGA	34	17 (34%)	DST	44	14 (28%)	ITPR1	26	13 (26%)
NBEAL2	34	17 (34%)	ELP1	20	14 (28%)	KAT6B	24	13 (26%)
NFAT5	22	17 (34%)	FCGBP	28	14 (28%)	KIAA1217	24	13 (26%)
PLXNA3	33	17 (34%)	FLNB	24	14 (28%)	KIAA1549	24	13 (26%)
PRKDC	35	17 (34%)	GREB1	18	14 (28%)	KIF13A	16	13 (26%)
RNF213	34	17 (34%)	GSE1	24	14 (28%)	KNL1	24	13 (26%)
SRRM2	36	17 (34%)	HCFC1	34	14 (28%)	LAMA3	28	13 (26%)
TRPM6	24	17 (34%)	HECTD1	22	14 (28%)	LOXHD1	26	13 (26%)
TRRAP	43	17 (34%)	HELZ2	19	14 (28%)	MAP2	20	13 (26%)
ZZEF1	35	17 (34%)	HERC2	32	14 (28%)	MED12	22	13 (26%)
ADGRG4	41	16 (32%)	HIVEP3	29	14 (28%)	MUC4	26	13 (26%)
ADGRV1	48	16 (32%)	ITPR2	23	14 (28%)	MYO16	26	13 (26%)
ANK2	49	16 (32%)	KAT6A	22	14 (28%)	MYO5A	18	13 (26%)
CACNA1B	25	16 (32%)	KIF1A	23	14 (28%)	MYO7A	27	13 (26%)
CFAP46	29	16 (32%)	KIF26A	19	14 (28%)	NCOR1	26	13 (26%)
DOCK10	24	16 (32%)	KMT2B	24	14 (28%)	NEXMIF	24	13 (26%)
DOCK2	22	16 (32%)	LMTK3	21	14 (28%)	NIPBL	28	13 (26%)
FAT3	44	16 (32%)	LTBP4	22	14 (28%)	NRAP	16	13 (26%)
FBN2	32	16 (32%)	MED1	18	14 (28%)	NUP133	16	13 (26%)
HMCN1	39	16 (32%)	MED13L	33	14 (28%)	NUP210	21	13 (26%)
HMCN2	41	16 (32%)	MEGF8	24	14 (28%)	NUP214	24	13 (26%)
HSPG2	39	16 (32%)	MN1	15	14 (28%)	OTOGL	21	13 (26%)
KMT2A	32	16 (32%)	MPRIP	19	14 (28%)	PCLO	34	13 (26%)
LAMA2	36	16 (32%)	MTOR	20	14 (28%)	PHKB	18	13 (26%)
LYST	32	16 (32%)	MUC17	45	14 (28%)	PHLPP1	22	13 (26%)
NSD1	35	16 (32%)	MXRA5	26	14 (28%)	PIEZO2	26	13 (26%)
OTOF	20	16 (32%)	MYO10	28	14 (28%)	PLXNB3	16	13 (26%)
POLE	24	16 (32%)	MYO15B	26	14 (28%)	PLXNC1	18	13 (26%)
RAI1	22	16 (32%)	NAV3	20	14 (28%)	PRAG1	17	13 (26%)
RYR2	40	16 (32%)	NBAS	27	14 (28%)	PRRC2C	24	13 (26%)
SBNO2	24	16 (32%)	NHS	23	14 (28%)	QRICH2	23	13 (26%)
SSPO	49	16 (32%)	NOTCH2	24	14 (28%)	RALGAPA2	21	13 (26%)
STARD9	38	16 (32%)	NOTCH4	23	14 (28%)	RAPGEF2	19	13 (26%)
TG	37	16 (32%)	PCNT	33	14 (28%)	RERE	18	13 (26%)
UNC80	34	16 (32%)	PDZD2	25	14 (28%)	RREB1	20	13 (26%)
ZAN	23	16 (32%)	PI4KA	20	14 (28%)	SAMD9	15	13 (26%)
ABCA13	38	15 (30%)	PRR12	23	14 (28%)	SDK1	23	13 (26%)
ABCA2	33	15 (30%)	PTPRF	27	14 (28%)	SETD2	23	13 (26%)

AKAP9	33	15 (30%)	REV3L	30	14 (28%)	SIPA1L3	21	13 (26%)
ANK3	33	15 (30%)	RSF1	16	14 (28%)	SMG1	27	13 (26%)
ARFGEF3	32	15 (30%)	SHANK3	19	14 (28%)	SPEG	19	13 (26%)
ATP2B3	18	15 (30%)	SOGA1	22	14 (28%)	SPTBN1	32	13 (26%)
BCOR	25	15 (30%)	SPTA1	25	14 (28%)	SPTBN2	22	13 (26%)
BIRC6	39	15 (30%)	TBCD	18	14 (28%)	SPTBN5	18	13 (26%)
CACNA1D	29	15 (30%)	TENM1	18	14 (28%)	SVEP1	18	13 (26%)
CNOT1	26	15 (30%)	TENM4	27	14 (28%)	TAF1	24	13 (26%)
COL11A1	32	15 (30%)	TRIP11	18	14 (28%)	TAOK2	16	13 (26%)
COL12A1	31	15 (30%)	USP24	31	14 (28%)	TCF20	21	13 (26%)
COL22A1	23	15 (30%)	UTRN	35	14 (28%)	THADA	20	13 (26%)
COL4A4	24	15 (30%)	VWF	34	14 (28%)	TLN1	17	13 (26%)
COL4A6	28	15 (30%)	ZFYVE26	26	14 (28%)	TLN2	23	13 (26%)
COL5A3	27	15 (30%)	ZNF236	23	14 (28%)	TNRC18	26	13 (26%)
CRAMP1	23	15 (30%)	A2ML1	18	13 (26%)	TRPM7	19	13 (26%)
CREBBP	24	15 (30%)	ABCC6	25	13 (26%)	TSHZ1	19	13 (26%)
CSMD2	30	15 (30%)	AFF2	19	13 (26%)	URB2	19	13 (26%)
DCHS1	30	15 (30%)	AHNAK	21	13 (26%)	VPS13B	32	13 (26%)
DEPDC5	25	15 (30%)	AHNAK2	56	13 (26%)	ZFHX2	21	13 (26%)
DNAH2	34	15 (30%)	ANKRD11	30	13 (26%)	ZNF106	27	13 (26%)
DYNC1H1	39	15 (30%)	ANKZF1	15	13 (26%)	ZNF628	16	13 (26%)

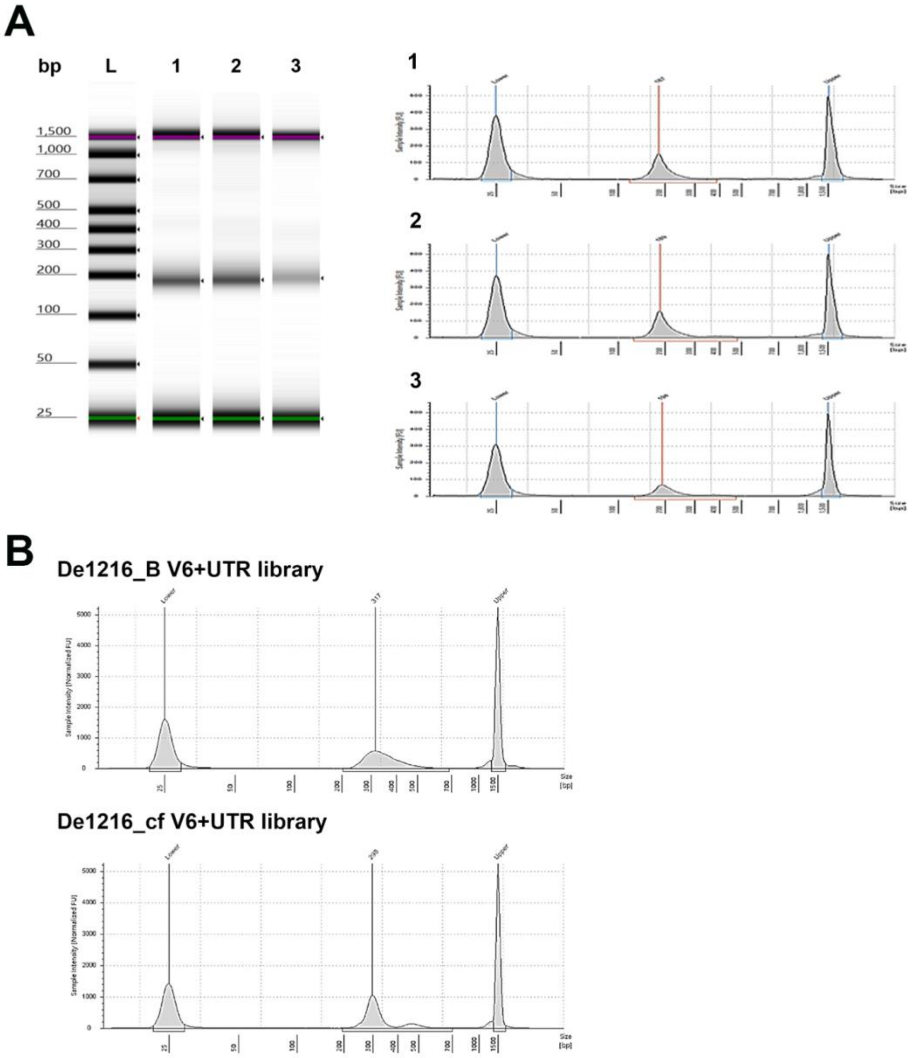

Figure S1. Agilent TapeStation analysis of the DNA integrity in cfDNA and matched normal samples. (A) Electropherogram and gel image of cfDNA and paired normal DNA in 50 patients with OSCC. (B) Representative electropherogram of DNA libraries sizes.

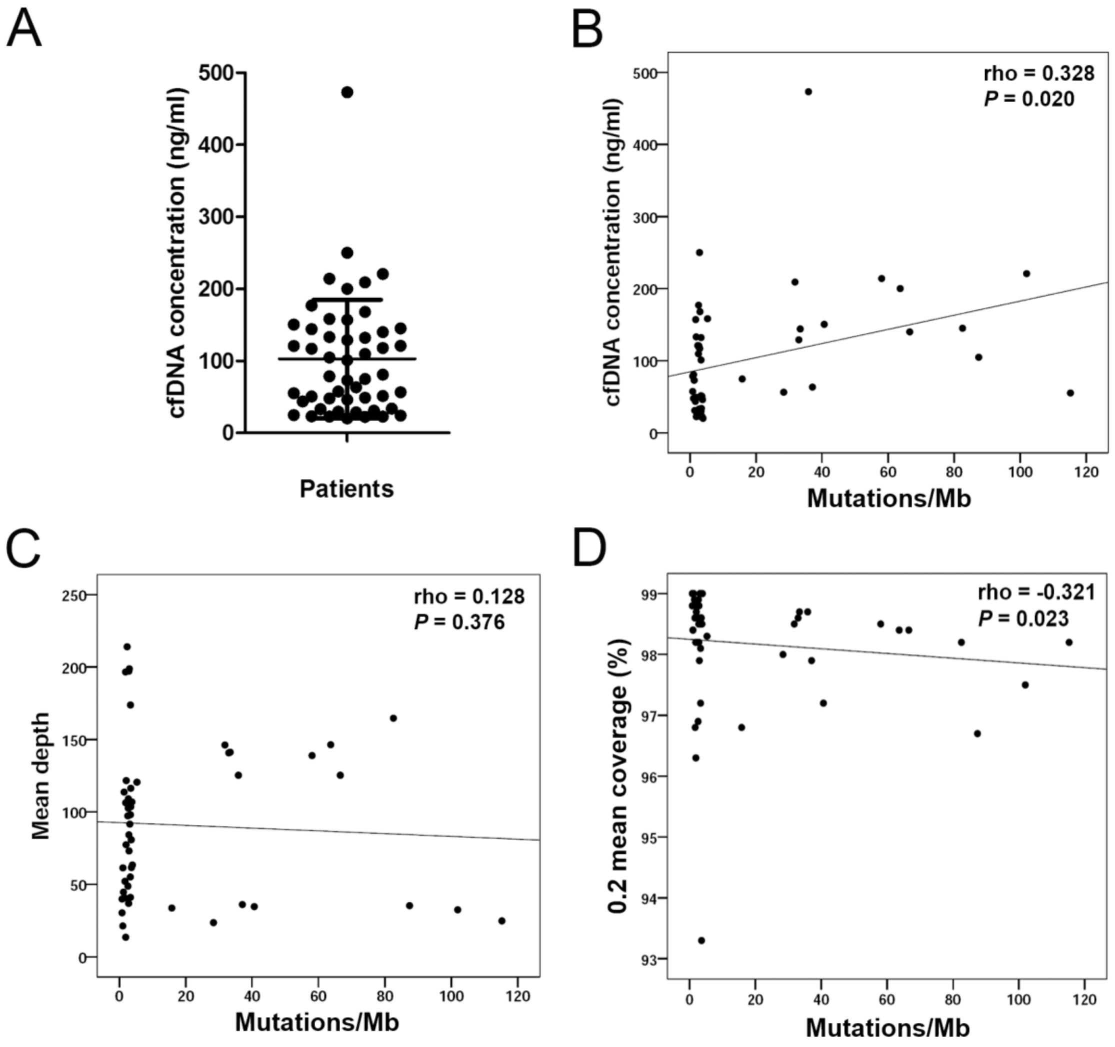

Figure S2. cfDNA mutation burden was correlated with cfDNA concentration, depth, and coverage. (A) Distribution of plasma cfDNA levels in patients with OSCC. cfDNA mutation burden was correlated with (B) cfDNA concentration, (C) mean depth, and (D) 0.2× mean coverage.

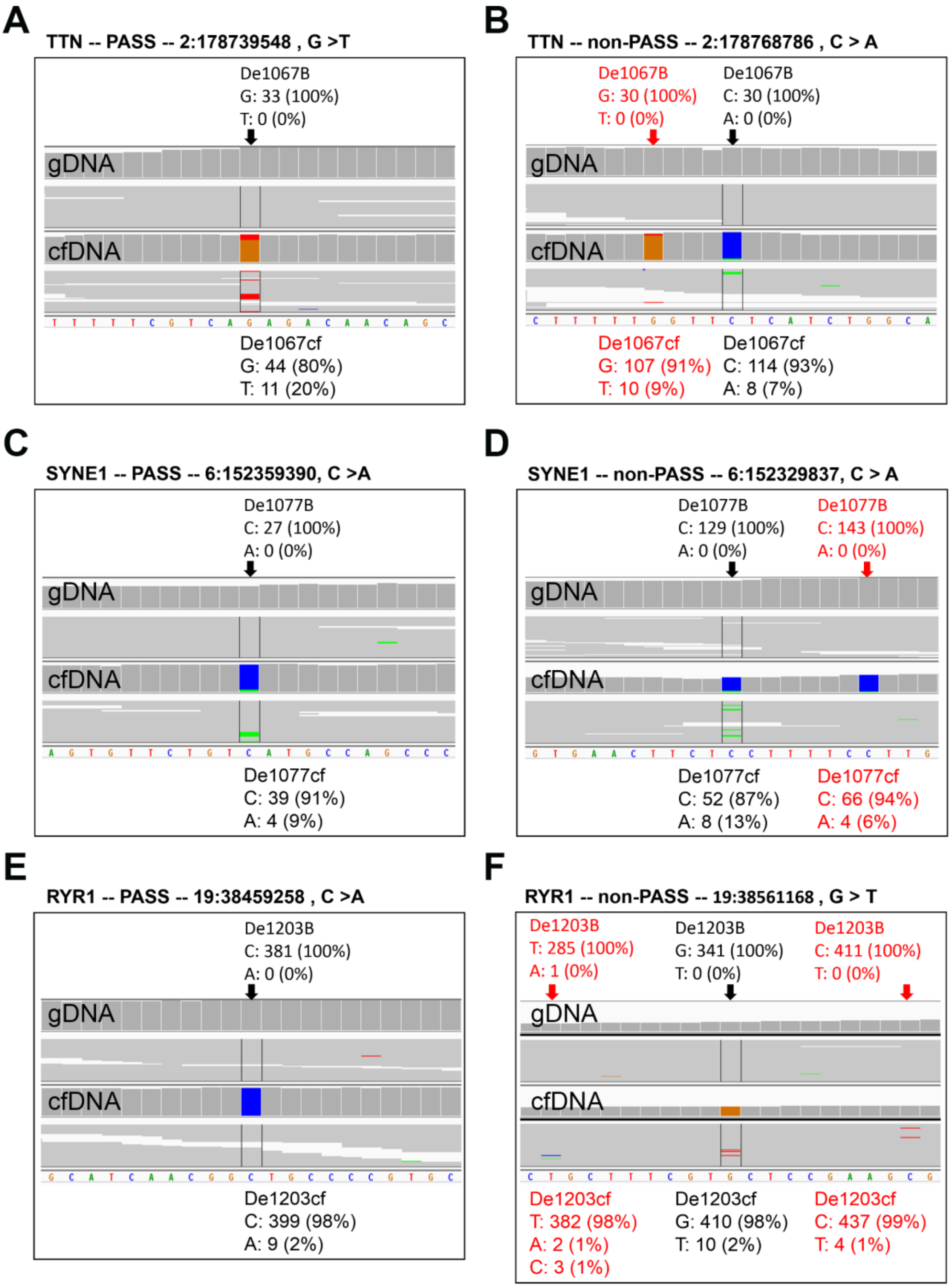

Figure S3. Visual verification of mutations in cfDNA using IGV. IGV PASS variants were detected in (A) *TNN*, (C) *SYNE2*, and (E) *RYR1*. IGV screenshot showing a variant with >2 mismatches within a 20 bp window in (B) *TNN*, (D) *SYNE2*, and (F) *RYR1*. These variants were considered false-positive mutations. Black arrows indicate the positions of the mutations. Red arrows indicate the positions of polymorphisms.

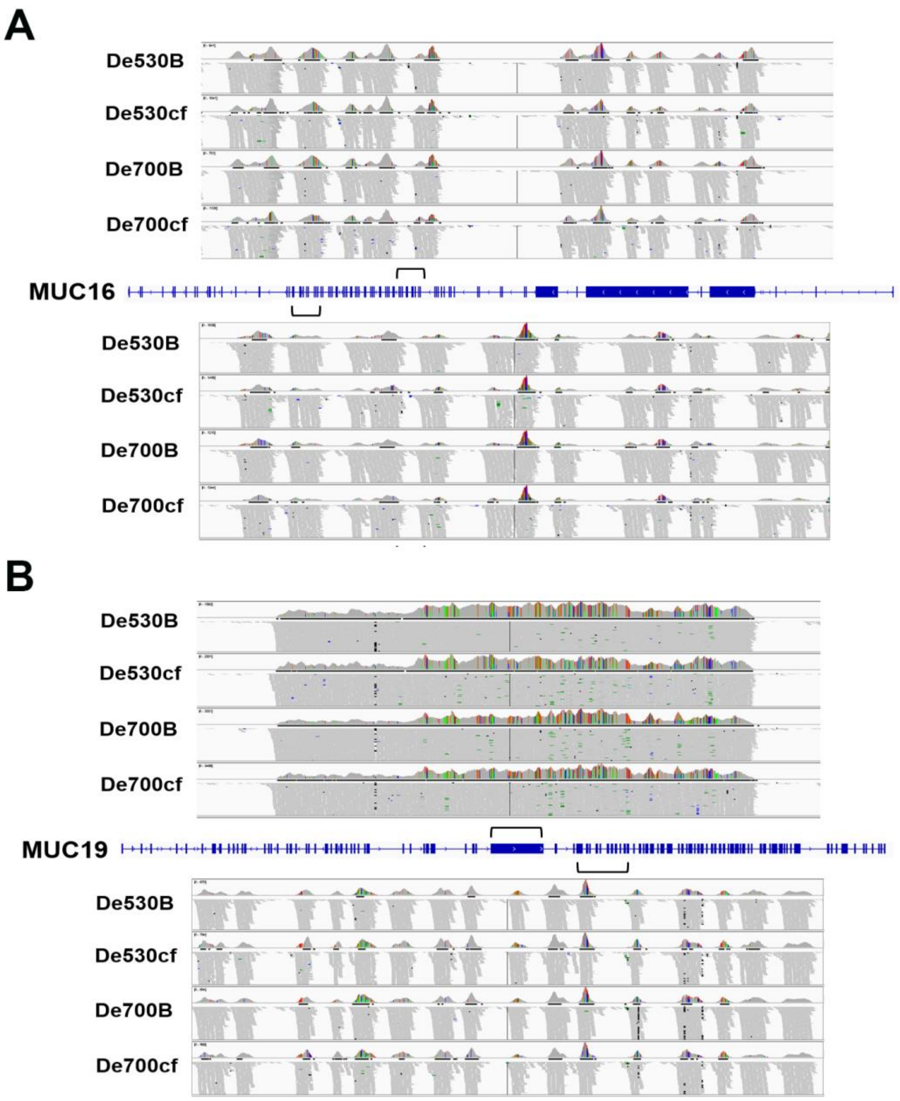

Figure S4. Recurrent false positives in *MUC16 and MUC19* were detected in cfDNA sequencing. IGV screenshots showthat many variants were detected in (A) the *MUC16* locusand(B) the *MUC19* locusin normal and tumor samples. Patients De530 and De700 are shown as representative patients, although other patients showed the same trend.

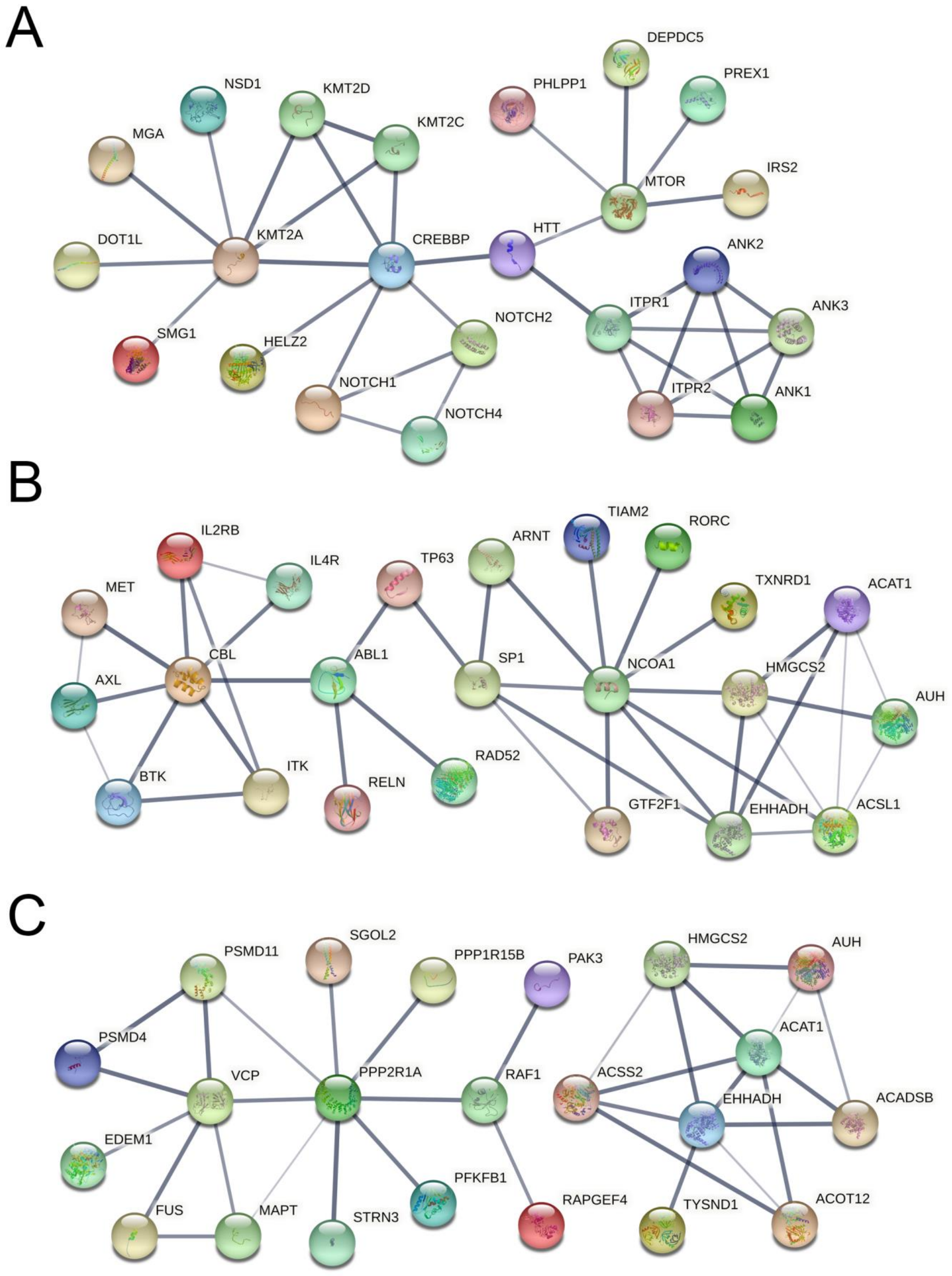

Figure S5. Simplified schematic representation of protein–protein interaction network analysis of mutated genes constructed using STRING. The interaction network was constructed based on the 300 most frequently mutated genes in (A) all patients, (B) patients with metastatic OSCC, and (C) expired patients. Line thickness indicates the strength of data support from text mining, experiments, databases, coexpression, neighborhood, gene fusion, and co-occurrence with a cutoff value of high confidence (0.7).

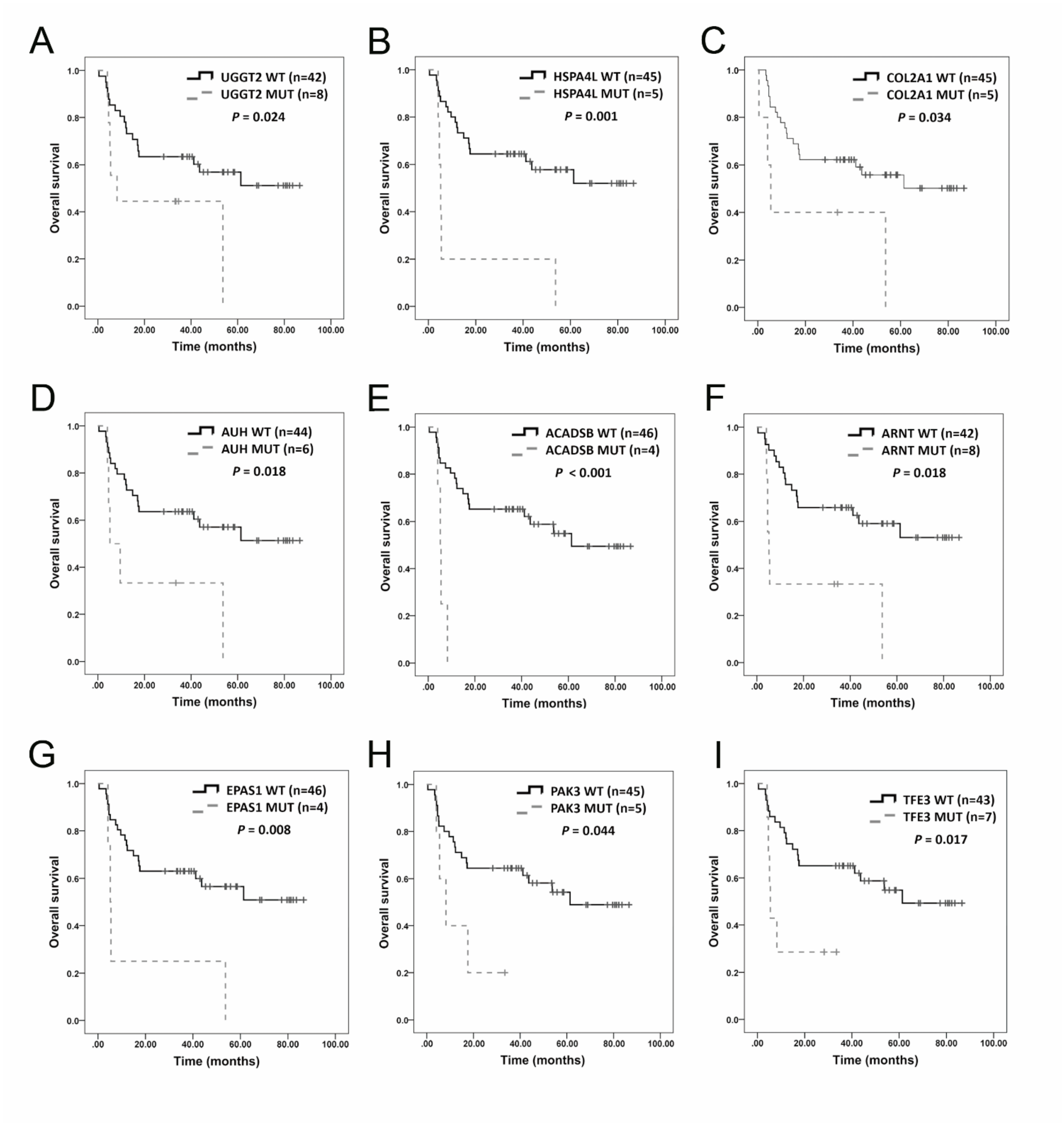

Figure S6. Survival analysis of candidate genes in patients with OSCC. Kaplan–Meier plots for overall survival in patients with metastatic OSCC based on (A) *UGGT2* mutation and (B) *HSPA4L* mutation status. Kaplan–Meier plots for overall survival in expired patients based on (C) *COL2A1*, (D) *AUH*, (E) *ACADSB*, (F) *ARNT*, (G) *EPAS*, (H) *PAK3*, and (I) *TFE3* mutation status. The Bonferroni-adjusted threshold for significance is set at $\alpha = 0.006$ (0.05/9).

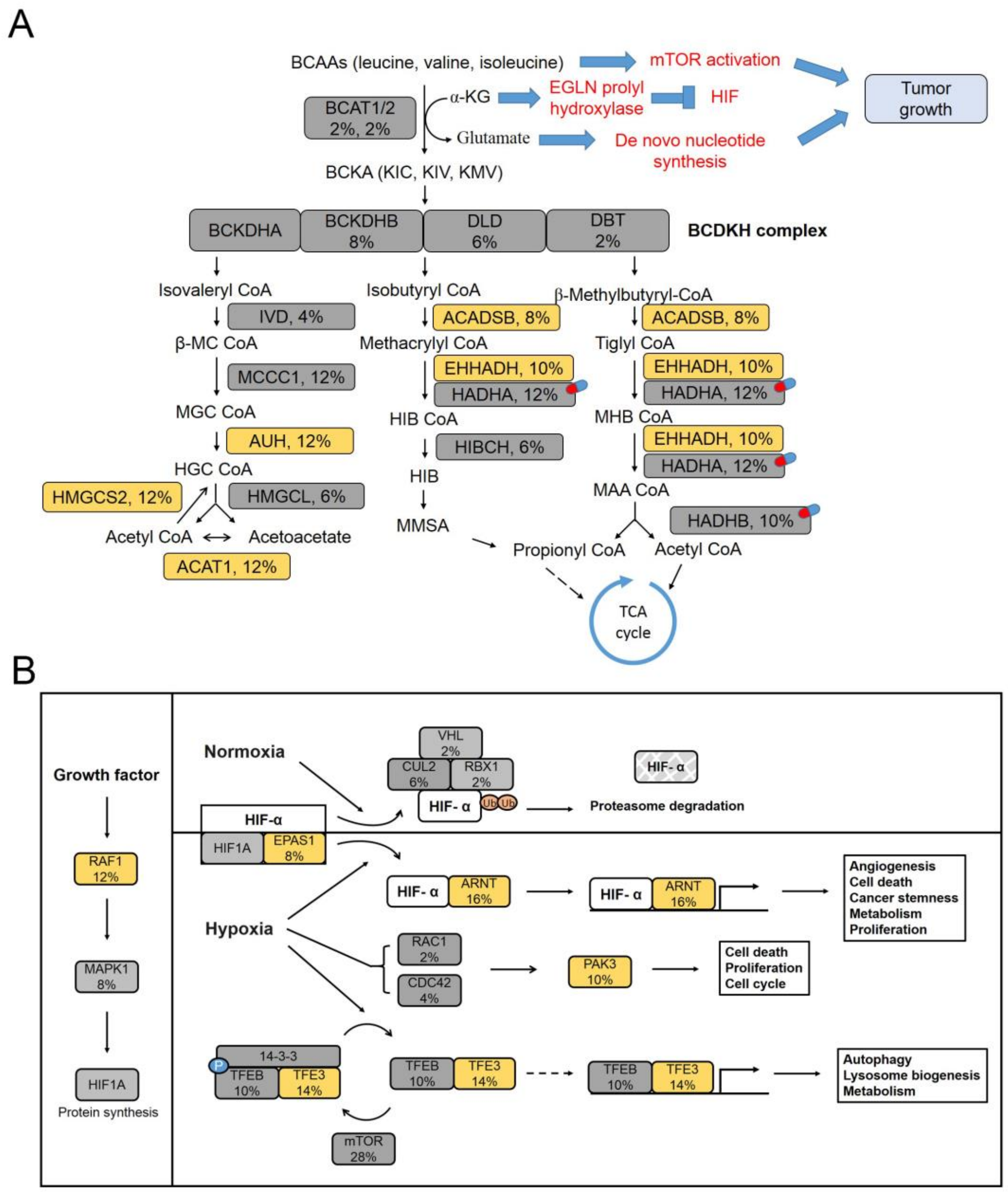

Figure S7. Mutation rates of the major altered genes in significantly altered pathways. The (A) BCAA catabolism and (B) hypoxia-related pathways were identified via KEGG pathway analysis in DAVID. Pill illustrations show FDA-approved drugs that target *HADHA* and *HADHB*. Genes were assigned to each pathway and are shown with their mutation frequencies (orange font: top 300frequently mutated genes in expired patients that were subjected to KEGG pathway analysis; gray font: genes were not subjected to KEGG pathway analysis).
